# The influence of maternal prenatal and early childhood nutrition and maternal prenatal stress on offspring immune system development and neurodevelopmental disorders

**DOI:** 10.3389/fnins.2013.00120

**Published:** 2013-07-31

**Authors:** Andrea Horvath Marques, Thomas G. O'Connor, Christine Roth, Ezra Susser, Anne-Lise Bjørke-Monsen

**Affiliations:** ^1^Department of Epidemiology, Mailman School of Public Health, Columbia UniversityNew York, NY, USA; ^2^Institute of Human Nutrition, Columbia UniversityNew York, NY, USA; ^3^Wynne Center for Family Research and Department of Psychiatry, University of Rochester Medical CenterNew York, NY, USA; ^4^Division of Epidemiology, Norwegian Institute of Public HealthOslo, Norway; ^5^Imprints Center for Genetic and Environmental Life Course Studies, Mailman School of Public Health, Columbia UniversityNew York, NY, USA; ^6^New York State Psychiatric InstituteNew York, NY, USA; ^7^The Dr. Lisa Oehler Visiting Professor, Department of Psychiatry, Göttingen UniversityGöttingen, Germany; ^8^Laboratory for Clinical Biochemistry, Haukeland University HospitalBergen, Norway

**Keywords:** programming, immunology, mental health, neurodevelopmental disorders, psychiatric disorders, nutrition, anxiety, stress

## Abstract

The developing immune system and central nervous system in the fetus and child are extremely sensitive to both exogenous and endogenous signals. Early immune system programming, leading to changes that can persist over the life course, has been suggested, and other evidence suggests that immune dysregulation in the early developing brain may play a role in neurodevelopmental disorders such as autism spectrum disorder and schizophrenia. The timing of immune dysregulation with respect to gestational age and neurologic development of the fetus may shape the elicited response. This creates a possible sensitive window of programming or vulnerability. This review will explore the effects of maternal prenatal and infant nutritional status (from conception until early childhood) as well as maternal prenatal stress and anxiety on early programming of immune function, and how this might influence neurodevelopment. We will describe fetal immune system development and maternal-fetal immune interactions to provide a better context for understanding the influence of nutrition and stress on the immune system. Finally, we will discuss the implications for prevention of neurodevelopmental disorders, with a focus on nutrition. Although certain micronutrient supplements have shown to both reduce the risk of neurodevelopmental disorders and enhance fetal immune development, we do not know whether their impact on immune development contributes to the preventive effect on neurodevelopmental disorders. Future studies are needed to elucidate this relationship, which may contribute to a better understanding of preventative mechanisms. Integrating studies of neurodevelopmental disorders and prenatal exposures with the simultaneous evaluation of neural and immune systems will shed light on mechanisms that underlie individual vulnerability or resilience to neurodevelopmental disorders and ultimately contribute to the development of primary preventions and early interventions.

## Introduction

The prenatal period is a sensitive time during which intrauterine exposures can modulate the course of development and confer an enduring effect on the offspring. Epidemiological and animal studies have demonstrated that prenatal programming of physiological systems can alter the growth and function of organ systems and pathology into adulthood (Szekeres-Bartho, 2002; Mold and McCune, 2012). For example, early immune system programming would give rise to changes in the fetal immune system that persists over the life course. In addition, some evidence based on animal, epidemiological and genetic studies suggests that immune dysregulation in the developing brain may play a role in neurodevelopmental disorders such as autism spectrum disorder and schizophrenia (Brown et al., 2000a, 2004; Susser et al., 2000; Meyer et al., 2009; Patterson, 2009, 2012; Meyer and Feldon, 2010; Bilbo and Schwarz, 2012; Aberg et al., 2013).

During pregnancy, the maternal and fetal immune systems communicate in a bi-directional manner. The maternal immune system develops an active immunologic tolerance against fetal-placenta antigens (recognition and activation). Following recognition, the maternal immune system reacts with a wide range of protective immunoregulatory mechanisms, which are critical for the maintenance of a normal pregnancy, the development of the fetal immune system, and maintaining maternal immunocompetence (Szekeres-Bartho, 2002; Mold and McCune, 2012; Erlebacher, 2013).

While well-controlled maternal immune responses play a positive physiological role in fetal immune and nervous system development, an inappropriate maternal immune activation (e.g., increased levels of pro-inflammatory cytokines) may contribute to an increased risk in the offspring of neurodevelopmental disorders, autoimmune diseases and allergies later in life (Brown et al., 2004; Bresnahan et al., 2005; Ellman and Susser, 2009; Bilbo and Schwarz, 2012). The timing of immune dysregulation, with respect to gestational age and neurologic development of the fetus, may be significant, as distinct immune and neurodevelopmental programs are affected differently depending on fetal stage. This creates a sensitive window of vulnerability (Dietert and Dietert, 2008).

The fetal immune system is particularly vulnerable to disruptions caused by environmental factors that have an impact on the maternal immune system, such as malnutrition, toxins and stress. We will discuss the effects of maternal and infant nutrition and maternal stress and anxiety on perinatal programming of immune function, and how this might influence neurodevelopment (Palmer, 2011; PrabhuDas et al., 2011; O'Connor et al., 2013b).

Based on these findings, we will discuss the implications for preventions of neurodevelopmental disorders focused on nutrition, as diet is among the more easily manipulated, safe, and promising avenues for intervention. We will highlight examples of three micronutrients—folate, iodine, and vitamin D—and discuss their proven and potential preventive effects on neurodevelopmental disorders. Although certain micronutrient supplements have shown to both reduce the risk of neurodevelopmental disorders and enhance fetal immune development, we do not know whether the impact on immune development contributes to the preventive effect on neurodevelopmental disorders. Future studies are needed to elucidate this relationship, which could contribute to better understanding of the mechanisms of prevention and in turn perhaps improvements in these interventions.

## Maternal-fetal immune system interactions

Maternal-fetal immune system interactions are characterized by bi-directional communications that rely upon the maternal immune system's recognition of antigens (derived from fetal, placental and paternal genomes) at the maternal-fetal interface, as well as a bi-directional transplacental trafficking of fetal and maternal cells throughout pregnancy (Taglauer et al., 2010; Mold and McCune, 2012; Nelson, 2012).

As the fetus is not in direct contact with maternal tissue, the placenta is the predominant source of antigens for T-cell priming and the direct target of any evoked maternal immune response. The maternal immune tolerance against fetal-placenta antigens depends on cell-cell interactions that take place between maternal immune cells resident in the decidua (natural killer cells and macrophages) and trophoblast antigens, as well as on immunoregulatory mechanisms (e.g., cytokines) that are critical for maintenance of a normal pregnancy, development of the fetal immune system, and maternal immunocompetence (Szekeres-Bartho, 2002; Mold and McCune, 2012; Erlebacher, 2013).

There are two main surfaces between the maternal and placental cells: (1) the placenta, located within the maternal decidua (a tissue layer derived from the endometrium) where placental trophoblasts and uterine leukocytes interact (Hanna et al., 2006; Erlebacher, 2013), and (2) the placental villi, where circulating maternal blood comes into contact with placental antigens, which can be shed directly into maternal circulation to modulate the maternal immune response (Hanna et al., 2006; Sargent et al., 2006; Erlebacher, 2013).

Fetal microchimerism (FMc) (i.e., the presence of fetal cells or DNA within maternal circulation and tissue) and maternal microchimerism (Mc) (i.e., the presence of maternal cells or DNA in the fetus) have been shown to persist long after birth (Bianchi et al., 1996; Maloney et al., 1999; Gammill and Nelson, 2010). FMc has been hypothesized to have both positive and negative effects on maternal cancer, endocrine and autoimmune diseases, but its role is still debated and controversial (Nelson, 1998; Feitsma et al., 2007; Fugazzola et al., 2011). FMc has been associated with several autoimmune disorders such as systemic sclerosis, juvenile dermatomyositis and juvenile diabetes; however, its role in these disorders still remains unclear (for review see Nelson et al., 2007; Gammill et al., 2010; Leveque and Khosrotehrani, 2011; Nelson, 2012).

### Development of maternal immune tolerance

#### Decidua natural killer cells, macrophages and trophoblast antigens

During the first trimester, the maternal decidua is mostly comprised of a unique type of natural killer cells called decidua natural killer cells (dNK), which are not present outside the context of pregnancy. The remaining maternal decidua is comprised of macrophages (CD 209^+^), while T-cells, dendritic cells and specialized B cells are scarce (Chazara et al., 2011; Erlebacher, 2013).

The primary role of dNK cells is to promote uterine vascular changes in the decidua in order to maximize maternal blood flow through the placenta. This process occurs by transforming the spiral arterioles into high-capacitance, low-resistance vessels, and by promoting trophoblast migration to replace the endothelium of these vessels with trophoblasts. The dNK-trophoblast interactions generate distant signals that trigger perivascular dNK cell and macrophage accumulation deeper within the decidua, further facilitating the vascular remodeling process of the arteriole (Hanna et al., 2006; Chazara et al., 2011; Erlebacher, 2013).

dNK cells can interact with trophoblast cells through two sets of receptors: (a) the killer-activating receptor, which recognizes a number of different molecules present on the surface of nucleated cells, and (b) the killer-inhibitory receptors (KIR family), which recognize the major histocompatibility complex (MHC) class I molecules that are typically present on all nucleated cells. When a killer-activating receptor is activated, a “kill” instruction is issued; however, this signal is normally overridden by the signal sent by the killer-inhibitory receptors that recognize MHC class I molecules. As all nucleated cells normally express MHC class I molecules on their surface, dNK cells will not destroy them. A lack of MHC class I molecules (due to infection or malignant transformation) leads to no inhibitory signal from the KIR and the consequent activation of dNK cells (Moretta et al., 1997; Delves and Roitt, 2000).

The dNK cell expresses a reduced cytotoxicity, which may play a role in protecting trophoblast cells from maternal anti-fetal responses. Some potential mechanisms have been suggested concerning the inhibition of dNK function, including (a) dNK interaction with anti-inflammatory cytokine IL-10, (b) dNK interaction with class Ib molecules (HLA-E or -G), and (c) a specific dNK-KIR coupled interaction with class Ia HLA-C2 alleles in the trophoblast, which leads to a strong activation of KIR and therefore inhibitory signal to dNK cells (for review see Mold and McCune, 2012; Erlebacher, 2013).

Macrophages in the decidua play an important role in tissue remodeling, maintenance of pregnancy, and parturition, but have also been associated with the development of preeclampsia and intrauterine growth restriction. Though much of the decidual macrophage biology remains unknown, these cells are skewed toward an M2-like macrophage phenotype, produce elevated levels of IL-10 and generate other factors that promote apoptosis, tissue remodeling, scavenging, debris clearance, and the generation of immunosuppressive tissue microenvironments (Heikkinen et al., 2003; Renaud and Graham, 2008; Erlebacher, 2013).

#### Maternal immunoregulatory mechanisms

The bi-directional relationship of the fetal-maternal immune response is also mediated by the production of cytokines (Breckler et al., 2008; Prescott et al., 2010). Maternal immune recognition of fetal antigens results in cytokine production that promotes blastocyst implantation and placenta growth, while embryogenic antigen expression on the placenta will modify maternal cytokine production (Breckler et al., 2008; Prescott et al., 2010).

An imbalance in maternal cytokine production can lead to fetal resorption (Wegmann et al., 1993; Tangri et al., 1994). In mice models, high doses of pro-inflammatory cytokines such as interferon (INF)-γ and tumor necrosis factor (TNF)-α [a T helper (Th)1 cytokine] have been associated with spontaneous abortion (Chaouat et al., 1990; Szekeres-Bartho, 2002). Conversely, healthy pregnancies and maternal immune tolerance are associated with a preponderance of Th2 cytokines (e.g., IL-10, IL-13), humoral immunity and reduced cell-mediated immunity (Th1-type cytokines) in response to both environmental antigens and fetal alloantigens (Raghupathy et al., 1999; Breckler et al., 2008, 2010).

Another important maternal immunoregulatory mechanism associated with pregnancy outcome is the balance between Th17 cells and the regulatory T-cells known as Tregs (originally termed suppressor T-cells). Tregs can suppress both Th1and Th2 responses (O'Garra and Vieira, 2004), while Th17 cells are important for the induction of autoimmunity and protection against infectious diseases. The regulation of the Tregs/T17 cell balance is critical in immunity and immunopathology and has been demonstrated to play an important role in maternal immune tolerance to fetal antigens and in fetal and neonatal immune development (Gustafsson et al., 2008; Prescott and Clifton, 2009). There is also evidence that the Treg (CD4^+^CD25^+^FoxP3^+^) regulatory response is initiated even prior to conception, following exposure to paternal antigens in seminal fluid (Robertson et al., 2009).

### Development of the fetal immune system and tolerance

Studies have shown that the fetal immune system actively responds to exogenous antigens such as infectious agents and vaccines (Marchant et al., 2003; Rastogi et al., 2007). However, in the absence of an additional signal, the fetal immune response tends toward an active tolerance rather than immunity (Munoz-Suano et al., 2011). This response pattern has important implications for understanding the impact of prenatal or early life exposure to infections or vaccines.

#### Development of the immune system: sensitive window of immune vulnerability

The development and maturation of the immune system starts early in fetal life and continues through infancy and early childhood. There is a period in which the immune cells have an increased vulnerability and susceptibility to environmental insults, such as malnutrition, stress and environmental contaminants. An early vulnerable window occurs when the tissues are being seeded by precursors of immune cells and varies depending on the type of immune cell (e.g., 4–7 weeks for myeloid-derived cells and 8–18 weeks for lymphoid cells) (Dietert and Dietert, 2008). However, there is no absolute consensus on the timeframe of the window of vulnerability, as both myeloid-derived cells and lymphoid cells continue to expand as gestation proceeds (Leibnitz, 2005). In addition, post-natal exposure (i.e., perinatal period and early childhood) to environmental insults has also been shown to modulate immune response (Suglia et al., 2010), but this topic is outside the scope of this review.

Myeloid-derived cells, such as macrophages, dendritic cells, skin Langerhans cells, and brain microglia cells, begin to populate the tissues during gestational week 4–7 and continue to increase in number throughout the second trimester (Leibnitz, 2005). The thymus is populated by lymphoid cells through the process of lymphocyte maturation and positive and negative selection (central tolerance) in gestational week 8–18, with continuing expansion of population within the thymus as gestation proceeds (Leibnitz, 2005). The first mature T-cells are seen in peripheral tissues in gestational week 10–12 and are circulating in significant number by the end of the second trimester (for review see Mold and McCune, 2012).

#### Development of immune tolerance

Although the mechanism underlying the development of tolerance in human beings remains unknown, some evidence suggests that it can be generated in response to maternal alloantigens that cross the placenta (for a detailed review see Mold and McCune, 2012).

During thymic maturation, potential self-reactive T-cells are deleted by a positive and negative central tolerance process. Positive tolerance refers to the process by which T-cells are exposed and bind (via T-cell receptors) to “self” MHC molecules. If T-cells fail to bind or bind with too high affinity to self-MHC molecules, then they are deleted (Griesemer et al., 2010). Following positive selection, thymocytes undergo negative selection, in which specialized epithelial cells and thymic antigen-presenting cells expose a broad range of self-peptides to T-cells. If T-cells respond to any of these antigens, they are deleted (Blackman et al., 1986). As a result, T-cells that bind to self-MHC molecules in conjunction with a foreign peptide, but do not recognize self-antigens, are selected. Disruption of the thymic maturation and clonal selection will influence not only the neonatal T-lymphocyte response, but also the likelihood of retaining auto-reactive T-lymphocytes later in life (Dietert and Piepenbrink, 2006).

Conversely, peripheral tolerance has been shown to play an important role in preventing autoimmunity and maintaining tolerance. It involves several types of regulatory T-cells (Tregs), although the best-characterized one is CD4^+^, which has high affinity receptors for IL-2 (CD25^+^) and the transcriptor factor Foxp3 (Hori et al., 2003). In humans, between the first and second trimester of gestation, there is an increased number of Tregs (CD4^+^CD25^+^), suggesting an active tolerogenic process (Cupedo et al., 2005). Tregs are generated either at the thymus by the process of T-cell selection, or at the peripheral tissue when naïve T-cells are activated in the presence of specific immunosuppressive factors (e.g., transforming growth factor (TGF)-ß) (Chen et al., 2003). It has been hypothesized that during development, expressions of high levels of TGF-ß in the fetal peripheral lymphonodus bias the fetal T-cell response toward a tolerogenic response, generating a large pool of fetal Tregs that are capable of recognizing and suppressing peripheral antigens, including maternal alloantigens (Mold and McCune, 2012).

#### Distinct features of fetal adaptive immune system associated with tolerance

Some evidence suggests that several distinct features of the fetal adaptive immune system play a role in the development of fetal tolerance against antigens that are present *in utero* (Mold and McCune, 2012). One process involves the presence of a specific fetal T-cell subpopulation called Vγ3δ5 T-cells, which are suggested to play a role in maintaining tissue homeostasis by regulating apoptosis and epidermal cell growth rather than by generating immunity to foreign antigens like adult α/ß T- cells (Sharp et al., 2005). Another mechanism includes the presence of a specific B-cell (IgM) subpopulation that is hypothesized to produce mature IgM cells that are broadly reactive, thus providing protection immediately following birth (Bhat et al., 1992).

Another unique feature of the fetal immune system is that fetal and neonatal T-cells and B-cells express auto-reactive antigen receptors that can also cross-react with peptides derived from unrelated antigens, providing a greater potential to respond to a broader range of infectious antigens, thus overcoming the limitations of having a smaller T-cell pool at birth (Gavin and Bevan, 1995; Mold and McCune, 2012).

Finally, fetal and adult hematopoietic stem cells (HSC) have a distinct phenotype and function, and are likely to generate different populations of mature hematopoietic cells (Ikuta et al., 1990) (for review Mold and McCune, 2012). While fetal HSCs are highly proliferative, undergo extensive self-renewal and are primarily maintained in the fetal liver, adult HSCs are relatively quiescent and mainly reside in the bone marrow. However, it is still not clear what mechanisms are involved in the transition from fetal HSC to adult HSC, or if fetal and adult HSC populations coexist during the fetal or neonatal period; studies have shown a dramatic shift in the turnover rates of hematopoietic cells between the first and second year of life (Rufer et al., 1999). These findings have important implications for understanding tolerance and immunity to infectious diseases, susceptibility to the development of atopic disease, and responses to vaccines during pregnancy and during the neonatal period.

#### The fetal immune system and central nervous system (CNS)

It is now well-established that the neurological and immune systems communicate with each other in a bi-directional manner. The CNS can regulate the immune system via both neuronal and hormonal pathways. Conversely, the immune system can affect the CNS either by local or peripheral processes (Marques-Deak et al., 2005; Silverman et al., 2005; Silverman and Sternberg, 2008; Dantzer, 2009; Marques et al., 2009; Thayer, 2009; Dantzer et al., 2011; Raison and Miller, 2011).

Although definitive pathways by which immune dysfunction can contribute to neurodevelopmental disorders are still not completely understood, the presence of maternal pathogenic autoantibodies, immune activation and increased levels of pro-inflammatory cytokines in the fetal brain can exert a negative impact on brain development if the time of exposure overlaps with major processes in neurodevelopment, such as cell migration, axonal elongation and dendritic tree maturation (Bilbo and Schwarz, 2009; Meyer et al., 2009; Patterson, 2011, 2012; Depino, 2013).

Because the blood-brain barrier (BBB) is not fully developed during the fetal period, larger molecules, such as antibodies, may have greater access to the brain (Diamond et al., 2009). BBB permeability increases as a result of microglia cell activation, infection, trauma or stress, and thereby enhances the risk of exposing the brain to insults and environmental stimuli that may impact neurodevelopment (Prat et al., 2001).

Maternal antibodies, transferred to the fetus by the placenta (starting as early as 6 weeks of gestation with a rapid increase by week 26–34) or to the infant through breast-feeding, play an important role in providing passive immunity to the fetus or neonate. However, the presence of pathogenic autoantibodies targeting the CNS in the developing fetus and neonate can be deleterious. It has been proposed that brain-specific maternal autoantibodies might underlie multiple congenital and neurodevelopmental disorders (for review see Billington, 1992; Lee et al., 2009; Fox et al., 2012).

Peripheral activation of the immune system with production of cytokines can affect the CNS indirectly (by activation of the hypothalamic-pituitary-adrenal (HPA) axis) or directly (via cytokines crossing the BBB (Silverman et al., 2005). In the CNS, microglia, astrocytes and other CNS cells produce and express receptors for cytokines in response to disturbances in homeostasis (Bilbo and Schwarz, 2009, 2012).

An imbalance between pro- and anti-inflammatory cytokines, favoring a pro-inflammatory response, has been associated with abnormal brain development and increased risk of neurodevelopmental disorders, including schizophrenia and autism (Brown et al., 2000a,b; Meyer et al., 2006, 2009; Deverman and Patterson, 2009; Boksa, 2010; Capuron and Miller, 2011; Bilbo and Schwarz, 2012; Depino, 2013). During normal brain development cytokines are expressed at very low levels, interact with all neural and non-neural cell types (e.g., microglia and endothelial cells) and play a role in brain development (for review see Deverman and Patterson, 2009). However, increased cytokine levels due to maternal immune activation (MIA) can potentially induce brain damage (Dammann and O'Shea, 2008; Deverman and Patterson, 2009; Patterson, 2009). Interestingly, an imbalance in cytokine expression (pro- *vs*. anti-inflammatory cytokines) may be even more relevant, as a shift toward an excess of anti-inflammatory cytokines has also been associated with abnormal brain and behavioral development (Dhabhar et al., 2009; Meyer et al., 2009).

MIA can be due to viral, bacterial or parasitic infections. Animal studies on MIA using mice and rats have demonstrated that early life exposure to infections or immune activation can lead to cognitive, behavioral or brain morphological abnormalities that are relevant to autism, schizophrenia and other psychosis-related disorders (Meyer and Feldon, 2010; Brown and Patterson, 2011; Hsiao and Patterson, 2011; Meyer et al., 2011; Patterson, 2011, 2012). Notably, long-lasting brain and behavioral abnormalities following MIA have also been demonstrated (Meyer and Feldon, 2010; Meyer et al., 2011; Garay et al., 2013).

MIA has also been described in human populations. Epidemiological studies have indicated that prenatal exposure to a wide variety of infections (e.g., influenza, rubella, toxoplasma gondii, etc.) is associated with an increased risk for schizophrenia and autism. For review see Susser et al. (2000); Brown et al. (2004); Bresnahan et al. (2005); Atladottir et al. (2010); Brown and Patterson (2011); Moore and Susser (2011).

### What would a prenatal programming influence on offspring immune function look like?

The complexity of the immune system means that, inevitably, no single study would be equipped to examine each of the multiple aspects of the functioning immune system, let alone the dynamic, systemic nature of its actions. For example, among those studies assessing variation in cytokines, the vast majority focuses on fewer than a handful of the multitude of cytokines identified. This could easily lead to misspecification of effects, given that cytokines are pleiotropic, can act in an antagonistic, synergistic or redundant manner with other cytokines, and have numerous possible functions in the body. The lesson is that it is no easy task to identify which aspects of the immune system might be affected by maternal prenatal nutrition factors and/or stress. Guidance for developing protocols for researching prenatal stress and anxiety in humans is available from animal studies and from the accelerating research activity on inflammation as an outcome of psychological risks and characteristics—although much of the work is still in the developmental and exploratory phases. What is clear is that general statements about prenatal malnutrition and stress/anxiety influencing the “immune system” are too broad and vague to be illuminating. Going forward, it will be important to clarify which specific aspects of immune function are predicted by prenatal risk factors, and the methods by which an association is demonstrated.

The notion that maternal prenatal malnutrition and stress/anxiety may alter some or many aspects of the developing immune system of the fetus/child is intriguing for several reasons. Perhaps the most obvious is that pregnancy is in itself a major immunological challenge for the mother and fetus. Indeed, alteration of the pregnant mother's immune system by the developing embryo is essential if the fetus is to be carried to term. Many of these changes have been well documented and were discussed in sections Decidua Natural Killer Cells, Macrophages and Trophoblast Antigens and Maternal Immunoregulatory Mechanisms. Some of these changes are captured by the alteration in autoimmune diseases in the course of pregnancy (Wilder, 1998). Any focus on *in utero* predictors would necessarily require some attention to the normative immune challenges created by the pregnancy.

If there were a prenatal programming mechanism for immune function in the child, then it is worth considering what such an effect would look like. One specific question is which components of the immune system might be programmable. As indicated above, the immune system is a complex array of moving and interconnected parts, and this confounds decisions about which markers to assess and what meaning the results might hold. As will be seen from the review of studies below, there are some trends in the operationalization and execution of immune system measurement. However, it is far from clear which aspects of the immune system might be especially susceptible to programming influence, or why that might be. A second, broader consideration is that it would not make much evolutionary sense for a species if its immune system—an essential feature for survival and reproductive fitness—could be “damaged” by a risk as potentially common as prenatal stress and anxiety. Therefore, rather than view immune outcomes in terms of simple “deficits,” it may be more accurate to view alterations in the immune system related to prenatal malnutrition and stress/anxiety as adaptations to early *in utero* exposures. The fundamental notion here is that the development of immunity in the fetus and infant is malleable and sensitive to input; the immune system “learns from” or responds to exposures. This model comports well with the adaptive response and carrying-forward of effects that is built into the immune programming hypothesis. In this context, it is interesting that at least some studies reporting a link between prenatal stress/anxiety and immune outcomes report alteration in the type of immune response (e.g., an exaggerated bias toward a type 2 response; see below) rather than merely reduced immune competence. Similarly, the link between prenatal stress/anxiety and asthma implies not a simple deficit in the immune system but an alteration in the development of the immune system that increases the likelihood of autoimmune diseases.

## The impact of maternal and infant nutritional status on immune system development and neurodevelopment

Maternal and infant nutrition may modulate the immunologic development of the fetus and young infant and permanently alter immunologic and regulatory mechanisms, which may affect the risk for later disease (Palmer, 2011). Adequate nutrition is necessary both for establishing the immune system, through normal organogenesis and development, and for an adequate immune response, through the normal proliferation of immune cells and the synthesis of secretory products and acute-phase proteins. Some nutrients, like antioxidants, are also needed to control or inhibit the immune response. Finally, the immune response in itself may affect nutritional status by decreasing levels of certain nutrients, exemplified by the decrease in iron, pyridoxine and tryptophan associated with inflammation (Lotto et al., 2011; Gaffney-Stomberg and McClung, 2012; von Bubnoff and Bieber, 2012).

### Maternal malnutrition

During pregnancy, maternal malnutrition hampers placentation, with resulting changes in placental size, morphology and blood flow (Belkacemi et al., 2010) that thereby reduce the supply of nutrients to the fetus. The subsequently compromised fetal nutrition status has profound effects on organogenesis, growth and fetal programming and has been associated with both short- and long-term effects on development and morbidity (Jansson and Powell, 2007).

Maternal malnutrition interferes with both the quality and quantity of immune factors transferred prenatally through the placenta and postnatally through the mammary gland (Palmer, 2011). Maternal IgG is actively transported through the placenta, appearing in the fetus (Simister, 2003) and remaining intact in the infant up to the age of 3–6 months (Palmer, 2011). The highest levels of maternal IgG in the cord blood is found during the last 4–6 weeks of pregnancy, so premature birth is associated with lower IgG levels in the infant (Malek et al., 1996). Reduced IgG levels have also been observed in small-for-gestational-age infants and in infants born to mothers with a lower than normal weight for height (Okoko, Wesumperuma, and Hart, 2001). In human milk the main antibody is IgA, which constitutes part of the mucosal immune system that controls epithelial colonization of microorganisms and inhibits penetration of harmful substances (Brandtzaeg, 2011).

### A window of opportunity

Development of oral tolerance depends on interactions between maternal, infant and environmental factors, including genetics, food and bacterial colonization. Breast milk is essential for development of oral tolerance and contains immunoglobulins, cytokines, growth factors, lysozyme, lactoferrin, and human milk oligosaccharides. Maternal and infant nutrition modulate immune system development by providing food antigens. Food-derived antigens are transferred through the placenta into the amniotic fluid and cause prenatal formation of antigen-specific IgE antibodies (Kondo et al., 1992). After birth, introduction of breast milk, formula and solid food influences immune maturation and response in a complex interaction with gut flora (Calder et al., 2006). There appears to be a window of opportunity for optimal development of oral tolerance between 4 and 6 months of age. Impaired oral tolerance is associated with gut inflammatory disease, food allergies and celiac disease (Verhasselt, 2010). Impaired oral tolerance was exemplified by an experience in Sweden between 1984 and 1996, during which an epidemic of coeliac disease in children <2 years occurred following changing of feeding habits and delaying introduction of gluten from 4 months to 6 months (Ivarsson et al., 2013).

### Micronutrients

Maternal deficiency of certain micronutrients is reported to permanently affect the immune system, but much remains unknown about this area. Gestational zinc deficiency has been associated with reduced thymic and spleen size, decreased antibody concentrations and impaired lymphocyte activity (Wellinghausen, 2001). Nutritional factors related to single carbon metabolisms, choline, folate (which is necessary for DNA methylation) and vitamins B2, B6, and B12 are important modulators for epigenetic mechanisms (Dominguez-Salas et al., 2012). Changes in the epigenetic regulation of immune system development have been linked to various pathological conditions, among them allergic risk and asthma development (Martino and Prescott, 2011). Dietary folate is postulated to be necessary for the maintenance of Forkhead box P3 (FOXP3) Tregs in the colon, a subset of inhibitory CD4+ helper T-cells that restrain the immune response to infection, inflammation and autoimmunity. Conversely, mice fed a diet deficient in folic acid have a higher susceptibility to intestinal inflammation (Kinoshita et al., 2012).

Fat-soluble vitamins A and D play important roles in both cell-mediated and humoral immune responses (Wintergerst et al., 2007). Vitamin D is a direct regulator of antimicrobial innate immune responses, can inhibit lymphocyte proliferation (Wang et al., 2004) and promote development of Tregs (Lemire et al., 1984). Vitamin D deficiency in healthy neonates has been linked to an increased risk of respiratory syncytial virus infection during the first year of life. Vitamin A has also been shown to be necessary for the development of de novo Tregs in the gut (Sun et al., 2007). Vitamin A deficiency has been reported to seriously affect hematopoiesis (Oren et al., 2003) and has been associated with a substantial increase in morbidity and mortality, especially due to diarrhea and measles in young infants (Fawzi et al., 1993).

It has also been suggested that maternal intake of vitamin E and polyunsaturated fatty acids during pregnancy may influence development of the immune system and be implicated in childhood asthma and allergy. Published reports, however, show conflicting results, and intervention studies are still lacking (Devereux, 2010).

### Environmental contaminants

Food not only provides nutrients but is also the most important source of environmental contaminants, specifically persistent organic pollutants (POPs). This is a group of highly resistant chemicals, including dioxins, furans, polychlorinated biphenyls and organochlorine pesticides, created by industrial activities and found in food, with the highest concentrations in fatty fish (primarily farmed salmon) (Schecter et al., 2010). These highly toxic compounds have been found to cross the placenta and to be excreted in breast milk (Fromme et al., 2010), thereby effectively clearing the amount of POPs in maternal tissue by up to 94% (Thomsen et al., 2010; Whitworth et al., 2012). The rapidly developing infant receiving this breast milk has immature organ systems and is especially vulnerable for toxic effects.

Early life exposure to various xenobiotics, including POPs, is associated with developmental immunotoxicity, which has been linked to neurodevelopmental disorders like autism spectrum disorders (for review see Dietert and Dietert, 2008). Epidemiological and human studies have demonstrated that there is a complex interaction between the immune system, environmental pollutants and neurodevelopment, although the exact mechanisms are still not well understood (Park et al., 2010). However, pre-, peri- and postnatal exposure to POPs is clearly associated with brain damage (Ribas-Fito et al., 2001; Walkowiak et al., 2001).

### Gut microbiota

Postnatally, infant nutrition, together with other factors like gestational age, mode of delivery, bacterial environment and use of antibiotics, also influences the composition of the gut microbiota. The enteric nervous system, which provides a bi-directional communication pathway between the brain, gastrointestinal cells and enteric microbes (termed the *brain-gut-enteric microbiota axis*), is important for immunological and neurological development, as well as later health. Interactions between microbes and gut mucosa cells foster immunological tolerance and recognition of pathogens, regulate the subsequent production of various pro- and anti-inflammatory cytokines, and stimulate Th17 cells and Toll-like receptors, which can differentiate between commensal and pathogenic bacteria and trigger immune processes.

The microbiota may also play a role in regulation of the HPA system. Animal studies in mice have observed an association between gut microbes and levels of brain-derived neurotrophic factor in the cortex and hippocampus, and related this to changes in brain plasticity (Sudo et al., 2004). Malnutrition has been also associated with stress and activation of the HPA axis (Seckl and Meaney, 2004). This is further outlined in the next section.

## The impact of maternal prenatal stress and anxiety on immune system development and neurodevelopment

Experimental animal studies in a very wide range of species demonstrate reliable links between prenatal stress and a wide range of outcome measures to a considerable degree. This research agenda has been systematically translated to human/clinical research programs in the past decade or so, with notable reported parallels (Talge et al., 2007; O'Donnell et al., 2009). Prenatal programming from maternal stress is now a significant line of investigation, with many active research programs assessing a wide range of outcomes and mechanisms.

One translation of the prenatal stress paradigm for human development that has only recently attracted attention is the association between prenatal stress and immune outcomes. Studies linking immune function with prenatal stress are notable for at least two reasons. The first is the possibility that prenatal stress or anxiety could influence the developmental programming for vulnerability to infectious disease. Second, such a link would further amplify the potential public health impact of maternal prenatal stress or anxiety. That is because clinically significant prenatal stress and anxiety is widely-reported to be common (Heron et al., 2004) and, moreover, disturbances in behavior and cognition linked to maternal prenatal stress and anxiety are not limited to the clinical extremes (Huizink et al., 2003). These observations compound the obvious public health relevance of infectious diseases in children. It is clear that there is variation in susceptibility to infectious disease; if this variation has prenatal origins, then there could be significant scope for prenatal preventive interventions as treatment.

### Empirical research linking prenatal stress to offspring immune outcomes

There is a growing empirical base supporting the hypothesis that prenatal stress and anxiety is associated with several different kinds of immune system alterations in the offspring/child; the most persuasive evidence derives from experimental animal studies.

### Experimental animal work

The hypothesis that prenatal stress alters the immune response in the animal has received wide-spread support in the rat, mouse, and other animal models (Kay et al., 1998; Merlot et al., 2008; Couret et al., 2009). For example, in a study of C57BL/6 male mice offspring of prenatally stressed dams, Diz-Chaves and colleagues (2013) found a variety of prenatal stress effects on immune responses, including increased expression of pro-inflammatory cytokines IL-1b and TNF-alpha in the hippocampus and TNF-alpha expression after Lipopolysaccharides (LPS) stimulation. These findings are notable for highlighting some of the parameters that need to be configured in studies, such as the potentially important distinction between observing the immune system markers during a resting state and response to an immunological challenge, and the location of the immune cell types that are targeted.

Questions about the usefulness of rat and mouse models for understanding inflammatory responses in humans have been raised based on a minimal overlap in genetic profile of the inflammatory response between the mouse and human (Seok et al., 2013). It is therefore important that there is also a sound body of research on prenatal stress and offspring immune response in a non-human primate model. In a series of studies, Coe and colleagues reported reliable links between prenatal stress and specific immune outcomes in infant and juvenile primates. For example, Coe (Coe et al., 1996) found that prenatal administration of adrenocorticotropic hormone (ACTH) (a precursor to cortisol, that might well be elevated by stress) in a Rhesus monkey model was associated with reduced T-suppressor cell activity in the offspring. In another report in the same species, they (Coe et al., 2002) linked prenatal stress to reduced levels of IL-6 and TNF-alpha in a stimulation model (with LPS) that may be accounted for by elevated HPA axis function in the stressed animal. These data suggest that HPA axis activity may be a likely cause of this diminished immune response, perhaps because of a higher set point for HPA axis activity. This and other possible mechanisms of effect are discussed below. The key observation at this point is that experimental non-human primate work, which has causal leverage not gained in human studies, shows reliable links between prenatal stress and several markers of immune competence in the juvenile and adult offspring.

### Human research linking prenatal stress/anxiety to immune outcomes in the child

A link between the experiences of, or exposure to, stress and reduced immune competence in children dates back many years (Meyer and Haggerty, 1962). Investigations into stress *in utero* and the child's immune system are, however, more recent. Support for a role of prenatal stress and anxiety and child immune function is suggested by several studies examining asthma (Lefevre et al., 2011; Khashan et al., 2012) and infectious disease (Nielsen et al., 2011) outcomes in children.

Perhaps the most direct test of the hypothesis that prenatal anxiety alters specific indicators of the developing immune system of the child was recently reported (O'Connor et al., 2013b). In that study, infants whose mothers were characterized as anxious in pregnancy, according to self-report and clinical evaluation, exhibited poorer adaptive immune response at 6 months of age. Altered or poorer adaptive immunity was measured from humoral immunity, indexed by reduced hepatitis B antibody titers following immunization (*in vivo*), and cell-mediated immunity, indexed by increased IL-4 and decreased IFN-γ responder cell frequencies to antigen (*in vitro*). The latter finding implies that there is a reduced type 1 and increased type 2 response in the infant; that is, consistent with other studies reviewed here, maternal prenatal anxiety exaggerates the normal type 2 skewing and less robust type 1 cytokine response to specific antigens (Ota et al., 2004; PrabhuDas et al., 2011). These predictions were independent of multiple confounds, including obstetric and psychosocial factors. Additionally, Entringer et al. (2008) used a retrospective design to examine prenatal stress and immune function in adult women. There was greater cytokine production of IL-4, IL-10, and IL-6 in peripheral blood mononuclear cells (PBMCs) in response to Phytohaemagglutinin (PHA) in adult women who reported that their mother experienced a stress in pregnancy compared to a non-prenatal stress group; there was no group difference in lymphocyte subpopulations that were assessed.

A separate series of studies links immune markers in cord blood with a broad index of stress. For example, perinatal stress indexed by mode of delivery was associated with lymphocyte subset distributions (Duijts et al., 2008). Also, Wright et al. (2010) reported that a composite index of prenatal stress was associated with increased IL-8 and TNF-α, increased IL-13 and decreased IFN-γ in cord blood mononuclear cells; these cytokine responses were observed in response to a range of stimuli, and the effects depended on which stimuli was used. Furthermore, cord blood immunoglobulin E (IgE) was linked with socio-economic disadvantage (Scirica et al., 2007), and IgE levels in the infant at birth were predicted by chronic stress as reported by mothers (Sternthal et al., 2009).

Collectively, the human data provide a strong suggestion that maternal prenatal stress and anxiety may alter aspects of the innate and adaptive immune systems in the child. In general terms, that parallels what has been reported in numerous animal models. However, there remains some uncertainty about how robust these associations in humans are, as there are few instances of replication—likely a consequence of the novelty of this area of research and the discrepant methods used across studies. At present, the strongest finding is that maternal prenatal stress and anxiety may exacerbate the type 2 response that is already present in the newborn. Functional significance of the research on specific immune markers is implied by survey studies associating prenatal anxiety or stress with specific illnesses, perhaps most notably asthma.

### Mechanisms of effect

The most basic question for mechanistic research is how maternal prenatal stress and anxiety is “communicated” to the fetus. A first consideration here is that the maternal prenatal risk phenotype seems to be somewhat broad, including stress (objective and subjective), anxiety (clinical elevations and continuous measures within the normal range), and possibly the broad construct of negative affect, which would also include depression; other constructs such as “pregnancy-specific” anxiety have been suggested. It is to be expected—but not yet demonstrated—that the prediction of child immune outcomes would not be particular to one maternal prenatal measure or construct. That means that mechanistic research needs to consider what biological risk may underlie these overlapping psychological constructs. In that regard, each of these psychological measures is thought to activate stress pathways, especially the HPA axis and the sympathetic nervous system; there is also a growing literature examining these psychological measures in relation to specific immune function, particularly inflammation. Importantly, each of these candidate biological systems in the pregnant women satisfies a key criterion: that the biological products of these systems pass through the placenta, can alter placental function, or predict fetal response (DiPietro et al., 2003; Glover et al., 2009; Monk et al., 2011; O'Donnell et al., 2012).

The most popular candidate mechanism so far involves alteration of the infant HPA axis in the child. This hypothesis is based on two key observations. The first is that prenatal stress, perhaps through alteration of the maternal HPA axis or the barrier enzyme 11βHSD2 (O'Donnell et al., 2012), may alter the function (e.g., set point, or reactivity) of the child's HPA axis. Evidence for this, which is soundly supported in experimental animal work in several species, derives from several studies of children (Van den Bergh et al., 2008; Davis et al., 2011; O'Connor et al., 2013a; O'Donnell et al., 2013), each using a different methodology. If the child's HPA axis function is altered to a higher set point or greater reactivity by maternal prenatal stress and anxiety, as implied by at least some of these studies, then that would predict a suppressed immune response in the infant given the well-documented immune-suppressive effects of glucocorticoids on immune function parameters, and inflammation in particular. Direct evidence for this connection is limited, but several supportive and suggestive findings have been reported, such as the weaker antibody titer response of pre-term infants who were given dexamethasone, a synthetic glucocorticoid, to prevent chronic lung disease (Robinson et al., 2004). In addition, thymus development is sensitive to corticosteroids during fetal development. Here again, however, it is important to provide specifics, as immune-suppressive effects of glucocorticoids may not be global but rather may be more evident on type 1 cellular immunity, leading to a shift toward type 2-mediated immune response (Elenkov, 2004). Interestingly, that model is consistent with much of the limited research on maternal prenatal stress and anxiety and child immune function reviewed above. And, more specifically, there was some suggestive evidence that prenatal anxiety is associate with reduced levels of IL-12, which is a main inducer of type 1 responses (O'Connor et al., 2013b).

An HPA axis-mediated effect is not the only possibility, however. If maternal prenatal stress and anxiety is associated with elevated inflammation, and the markers of inflammation (e.g., pro-inflammatory cytokines) cross the placenta/alter placental function (which they do), then the fetus of prenatally stressed and anxious women might then be exposed to elevated immune responses early in development. Evidence supporting a link between maternal anxiety, stress and depression and pro-inflammatory cytokines in pregnancy has been reported in some (Coussons-Read et al., 2007) but not other studies (Blackmore et al., 2011), so the strength of this model is not yet clear. These studies imply inconsistent evidence with specific immune markers; there is, on the other hand, a sizable literature showing that maternal prenatal stress alters obstetric outcomes. Thus, maternal stress has been linked with increased risk of miscarriage (Neugebauer et al., 1996; Nepomnaschy et al., 2006), reproductive tract infection (Culhane et al., 2001), and younger gestational age at birth (Wadhwa et al., 1993). The presumption is that this may be at least partly because of increased inflammation that endangers the pregnancy, an association first demonstrated many years ago (Harris, 1919). Other studies show that manipulating the maternal immune system prenatally (through antibiotic use) may alter the fetal/child immune system, leading in one instance to an increased risk of asthma (Stensballe et al., 2012). Thus, a prenatal programming mechanism might operate through immunological changes in the mother that are then transferred more or less directly to the developing fetus.

A further possibility is that morphological changes induced by prenatal stress affect brain regions central to both HPA axis and immune activity. For example, in research on several species, animals exposed to prenatal stress show reductions in hippocampal volume (Lemaire et al., 2000; Coulon et al., 2013). Human studies on prenatal stress and brain morphology relevant to stress response or immune functioning are now underway in several labs; limited reported evidence so far does not suggest a link between prenatal stress and reduced hippocampal volume in children (Buss et al., 2012).

Challenges to each of these mechanisms abound. The human research has not adequately addressed important confounds ranging from nutrition (Monk et al., 2012) to genetics. In the case of genetics, for example, many functional polymorphisms of the glucocorticoid receptor (GR), mineralocorticoid receptor (MR), corticotropin-releasing hormone (CRH), serotonin or other genes may modulate the link between prenatal anxiety and child outcomes, with one example with infant temperament so far reported (Pluess et al., 2011), although there are large-scale studies failing to replicate (Braithwaite et al., 2013), and so this question remains unresolved. Additionally, the role of postnatal rearing conditions, which has been studied extensively in several animal species, e.g., non-human primates (Lubach et al., 1995), has not yet been studied in relation to immune outcomes in children. The possibility that caregiving may moderate the effects of prenatal anxiety on other outcomes including behavioral and cognitive development has been demonstrated (Bergman et al., 2010).

Mechanistic studies are also hampered by an under-developed model of psychoneuroimmunology. For example, we still lack basic information on how the developing immune system in the child responds in the short- and long-term, to brief or chronic stress exposure. Much is known about the immune system response to stress in the adult (perhaps especially the aged adult), but it is far from clear if parallel responses are found in children, or at what stage in development parallel links between stress exposure and immune system response become robust. That is because of the limited research on stress and immune system development in pediatric samples, (e.g., see Caserta et al., 2008, 2011; Low et al., 2013). And, clearly there will be some difficulty in differentiating among “directions of effect” in inherently bi-directional and mutually influencing systems, such as the stress response and immune systems. So, for example, elevated prenatal pro-inflammatory cytokines such as IL-1, IL-6, and TNF-alpha may induce production of maternal prenatal glucocorticoids through their action on the HPA axis, and this may then lead to altered stress and immune function development in the child. Recognizing the inter-dependent nature of the stress and immune system development means that, for example, targeting the stress response may have salutary effects on the immune system and vice versa.

Good immune function is critically important for babies, who must develop the cellular machinery to respond to immune challenges during fetal development but are nevertheless immunologically immature at birth; furthermore, this pressure to mount immune responses is intensified by an intensive vaccination schedule. Not surprisingly, then, there is substantial interest in how well babies' immune systems respond to early challenges, and what factors mediate individual differences. Understanding how immune function development may be influenced by maternal prenatal stress or anxiety is therefore important in its own right. Moreover, understanding immune system challenge and development might help explain some of the other neurodevelopmental outcomes thought to be causally linked with maternal prenatal stress or anxiety. For example, as previously discussed, there are clear linkages in animal studies (i.e., mouse and rat) between cytokines levels and brain development (Meyer et al., 2006; Patterson, 2009; Bilbo and Schwarz, 2012), and this pathway explains some of the associations described linking maternal viral or bacterial infection in pregnancy with neuropsychiatric disorders (Susser et al., 2000; Brown et al., 2004, 2000b).

## Implications for prevention of neurodevelopmental disorder focusing on nutrition

We emphasized in previous sections that optimal development of the fetal immune system depends on adequate maternal nutritional intake and that certain micronutrients play a particularly important role. We also noted that the development of the immune system may modulate the risk for later diseases, including neurodevelopmental disorders. Taken together, these observations raise the question of whether nutritional interventions, and in particular, micronutrient supplements (to mother or baby), could reduce the risk of neurodevelopmental disorders by enhancing early development of the immune system.

Thus far, this question has rarely been addressed directly. We have learned that certain micronutrient supplements both reduce the risk of neurodevelopmental disorders and enhance fetal immune development. But we do not know the relationship between these two effects, or whether the impact of supplements on immune development also contributes to the preventive effect. A useful focus for future research would be to elucidate that relationship, which could lead to a better understanding of the mechanisms of prevention and in turn, perhaps improvements in these interventions. From a global perspective, this is quite a fundamental question for public health, because severe micronutrient deficiencies are widespread in developing countries, and are associated with low birth weight, delayed neurodevelopment, stillbirths, and perinatal and neonatal mortality (Ahmed et al., 2012). The immunological development of the fetus or infant may well play a role in vulnerability to these adverse outcomes (Trehan et al., 2013).

There are several examples of broad nutritional interventions (e.g., encouragement of breastfeeding), as well as many examples of specific micronutrient supplements that fall within this framework (that is, they enhance both immune development and neurodevelopment but the relation between these two effects remains unknown. For illustration, we have selected three micronutrients—folate, iodine, and vitamin D—and discuss their proven and potential preventive effects on neurodevelopmental disorders.

### Folate

In an earlier section we described how folate and other B-vitamins can be pivotal for fetal immune development. With respect to neurodevelopmental disorders, periconceptional folic acid supplements have been proven to have a major preventive effect. Worldwide, birth defects affect about 6% of live births (Wallingford et al., 2013). Neural tube defects (NTDs) are one of the most common congenital birth defects and one of the few for which we have knowledge about preventive strategies. Folic acid is a B-vitamin that is important for cell proliferation, (Zeisel, 2009) central nervous system cell repair, (Iskandar et al., 2010) and appropriate epigenetic expression of the genome (Jaenisch and Bird, 2003; Steegers-Theunissen et al., 2009), as well as for immune development (Kjer-Nielsen et al., 2012). Randomized controlled trials have demonstrated that periconceptional folic acid supplements reduce the risk of NTDs (Prevention of neural tube defects: results of the Medical Research Council Vitamin Study, MRC Vitamin Study Research Group, 1991; Czeizel and Dudas, 1992; Berry et al., 1999). All women of childbearing age are recommended to take a folic acid supplement of 400–800 μg/d, preferably a month before conceiving. In addition to public health campaigns to increase awareness of taking prenatal folic acid supplements, the US Food and Drug Administration mandated adding folic acid to all enriched cereal grain products in 1998. This fortification policy has been associated with a prevalence decline of 34% for spina bifida and 20% for anencephaly (Honein et al., 2001; Williams et al., 2002; Canfield et al., 2005). Somewhat greater declines are reported for Canada (De Wals et al., 2007) and Chile (Lopez-Camelo et al., 2005). Early detection through prenatal screening is also a likely contributor to lower prevalence; prenatal ultrasound at gestational week 18–20 detects structural anomalies in about 60% of cases (Gagnon et al., 2009).

Recently, evidence is growing that periconceptional folic acid supplements may have preventive effects on other neurodevelopmental disorders as well. These recent studies can be traced in part to an intriguing finding from the Dutch Famine studies. The Dutch Hunger Winter was a period of severe starvation in West Holland toward the end of the Second World War. Gestational timing of starvation could be estimated because severe famine was relatively brief and food supply was abruptly restored when liberation arrived in May 1945. It was shown that the offspring of women with periconceptional exposure to severe famine—approximately one month before to two months after conception—had an increased risk not only of NTDs, but also of schizoid personality disorder at age 18 and schizophrenia in adulthood (Susser et al., 1996, 1998; Brown and Susser, 2008; Susser and St Clair, 2013). This coincident increase in NTDs and other neurodevelopmental disorders naturally led to the question of whether folic acid supplements could have broader preventive effects. Several studies now support this view, although results are not definitive enough as a basis for intervention. These include results from a large Norwegian prospective pregnancy cohort, where use of prenatal folic acid supplements around the time of conception has been associated with a lower risk of severe language delay (Roth et al., 2011) and autistic disorder (Surén et al., 2012), and a reduced risk of autism spectrum disorders was also observed in a case-control study in California (Schmidt et al., 2012).

### Iodine

Iodine deficiency is the leading preventable cause of mental retardation worldwide, and women of childbearing age and infants are at particular risk (Trumpff et al., 2013). Iodine is a micronutrient necessary for the production of thyroid hormones. Thyroid hormones play an essential role in the central nervous system during fetal and early postnatal life. Since iodine was not discussed in the earlier section on fetal immune development, we note that increasing evidence suggests that the immune response is modulated by thyroid hormones (De Vito et al., 2011).

About two billion people ingest too little iodine, and even moderate deficiency is known to lower intelligence. Prevention of iodine deficiency is easily managed by spraying regular table salt with a potassium iodate solution at very low costs. Iodized salts have been a true public health success story, with a rise in the world's household consumed iodized salt increasing from 25% in 1990 to 66% in 2006. Even so, it is presently a concern that iodine deficiency still exists in some regions of the world and has reappeared in some European countries (Vanderpump et al., 2011; Brantsaeter et al., 2013). The World Health Organization recently raised their recommended dietary iodine intake in pregnancy from 200 to 250 μg/d (Zimmermann, 2009).

### Vitamin D

We have described earlier how vitamin D is pivotal in early immune development and it is also known to be crucial for early skeletal development. It has been proposed, though not proven, that low levels of vitamin D during pregnancy and infancy could increase the risk of neurodevelopmental disorders, and that supplementation could play a role in prevention. An intriguing body of work comprising both rodents and human studies currently supports this view, though no definitive conclusions have been reached yet (Eyles et al., 2013; Pludowski et al., 2013; Yang et al., 2013). Notably, in a recent population-based case control study of 424 individuals with schizophrenia and 424 matched controls, dried blood spots taken at birth were tested for vitamin D levels (McGrath et al., 2010). When vitamin D levels were divided into quintiles, the investigators observed an increased risk of schizophrenia in individuals with vitamin D levels in the three lowest quintiles compared to the fourth quintile. Surprisingly, they also observed an increased risk for schizophrenia in individuals with vitamin D levels in the highest quintile.

Notably, breastfed infants are at especially high risk of vitamin D deficiency due to poor penetrance of vitamin D metabolites in milk (Kovacs, 2013; Thiele et al., 2013). The average level of vitamin D in breast milk is typically 25 IU per liter or less. Fish oil (that commonly contains vitamin D) and other Omega 3 fatty acid supplements taken in infancy have been associated with improved cognitive development, though this evidence is also far from definitive (Auestad et al., 2003; Karr et al., 2011; Meldrum et al., 2012; Luchtman and Song, 2013). The American Academy of Pediatrics recommends that all breastfed infants receive a daily vitamin D supplement of 400 IU, beginning in the first few days of life (Wagner et al., 2008).

## Conclusion

Early immune system programming can give rise to changes in the fetal immune system that can persist over the life course. Evidence based on animal, epidemiological and genetic studies suggests that immune dysregulation in the developing brain may play a role in neurodevelopmental disorders, such as autism spectrum disorders and schizophrenia.

The interface between the maternal and fetal compartments plays a central role in how the maternal immune system influences fetal and placental development. The maternal immune system develops an active immune tolerance against fetal-placenta antigens that arises from cell-cell interactions taking place between maternal immune cells resident in the decidua and trophoblast antigens. A range of protective immunoregulatory mechanisms is also critical for the maintenance of a normal pregnancy, development of the fetal immune system, and maternal immunocompetence. The development of the fetal immune system is characterized by the presence of several distinct features that promote fetal active tolerance against both *in utero* maternal antigens and exogenous antigens, such as infectious agents, vaccines and food antigens. This response pattern has important implications for understanding the impact of prenatal and early life exposure to infections, vaccines, and maternal immune activation.

While well-controlled maternal immune responses play a positive physiological role in fetal immune and nervous system development, an inappropriate maternal immune activation may contribute to an increased risk in the offspring of neurodevelopmental disorders, autoimmune diseases and allergies later in life. Disruption of the fetal immune system can affect the CNS either by local or peripheral processes. Although the pathways by which immune dysfunction can contribute to neurodevelopmental disorders are still not completely understood, the presence of maternal antibodies, immune activation (maternal and fetal) and imbalance of cytokine expression (pro- *vs*. anti-inflammatory) in the fetal brain can exert a negative impact on brain development if the time of exposure overlaps with major processes in neurodevelopment, such as cell migration, axonal elongation and dendritic tree maturation. Animal studies on maternal immune activation have demonstrated that early life exposure to infections and other factors that lead to immune activation may impose cognitive, behavior and brain morphological abnormalities analogous to findings described in patients with autism, schizophrenia and other psychosis related disorders. These results are supported by epidemiological studies linking maternal prenatal exposure to a wide variety of infections (e.g., influenza, rubella, toxoplasma gondii, etc.) with an increased risk for schizophrenia.

The fetal immune system is particularly vulnerable to environmental insults (e.g., malnutrition, toxins, infections, and stress), mainly during periods when tissue is seeded by precursors of immune cells, which varies depending on the cell type (sensitive window of immune vulnerability). However, deviations in the maternal or fetal immune systems associated with prenatal exposures should be interpreted with caution, as these may be the result of healthy and normal adaptation responses to pregnancy.

Adequate maternal/fetal nutrition is also necessary for the development of fetal and neonatal immune responses and immune cell proliferation, placentation, and the development of oral tolerance. Maternal malnutrition can reduce the supply of nutrients and immune factors to the fetus (via the placenta) and to the infant (via the mammary gland). In particular, maternal micronutrient deficiencies in zinc, fat-soluble vitamins (e.g., vitamins A, D, E) and nutritional factors related to single carbon metabolism (e.g., choline, vitamins B2, B6, B12 and folate) have been shown to play a role in cell-mediated and humoral immune responses. They also have been associated with increased offspring risk for respiratory infections (vitamin D), intestinal inflammation and diarrhea (folate and vitamin A), allergy and asthma (vitamin E and folate) and neurodevelopmental disorders (folate). In addition, food-derived antigens have also been shown to play a role in the development of the fetal immune system (prenatal formation of antigen specific IgE), infant immune system and brain development (oral tolerance and brain-gut-enteric microbiota axis). Prenatal stress and anxiety may also alter aspects of the offspring's innate and adaptive immune systems. However, uncertainty remains about how robust these associations are in humans; the strongest finding is that maternal prenatal stress and anxiety may exacerbate type 2 immune responses already present in the newborn. It has been hypothesized that the impact of maternal prenatal stress and anxiety on the fetal immune system might operate through alteration of the maternal HPA axis, ultimately altering the function (e.g., set point, reactivity) of the child's HPA axis, with a higher set point or greater reactivity of the infant HPA axis predicting a suppressed immune response. Another hypothesis is that immunological changes in the mother associated with maternal prenatal stress and anxiety (e.g., pro-inflammatory cytokines) could cross the placenta and/or alter placental function, consequently exposing the fetus to an elevated immune response early in development.

Interestingly, several examples of nutritional interventions (e.g., encouragement of breastfeeding and specific micronutrient supplements) have been shown to enhance both neurodevelopment and immune development. However, it is still not clear whether nutritional interventions, and, in particular micronutrient supplements (to mother or baby), could reduce the risk of neurodevelopmental disorders by enhancing early development of the immune system. We reported three examples of micronutrients—folate, iodine, and vitamin D—and discussed their proven and potential preventive effects on neurodevelopmental disorders. Future studies are needed to elucidate this relationship, which could contribute to a better understanding of preventive mechanisms and, in turn, perhaps improvements in these interventions.

Based on these findings, we suggest that integrating studies of neurodevelopmental disorders and prenatal exposures (e.g., nutrition and stress) with simultaneous and precise neural and immune system measures can potentially shed light on immunological mechanisms that underlie individual vulnerability or resilience to neurodevelopmental disorders. This research could ultimately contribute to the development of primary preventions and early interventions for mental disorders.

### Conflict of interest statement

The authors declare that the research was conducted in the absence of any commercial or financial relationships that could be construed as a potential conflict of interest.
